# Effect of systemic isotretinoin therapy on semen parameters

**DOI:** 10.1080/07853890.2023.2207038

**Published:** 2023-05-10

**Authors:** Abdullah Gurel, Gulhan Gurel, Fatma Fırat, Esra Ozgul, Irem Nur Durusu Turkoglu, Tugce Aladag, Ibrahim Baran Duran, Burhan Baylan

**Affiliations:** a Department of Urology, Afyonkarahisar Health Sciences University, Afyon, Turkey; b Department of Dermatology, Afyonkarahisar Health Sciences University, Afyon, Turkey; c Department of Histology & Embryology, Afyonkarahisar Health Sciences University, Afyon, Turkey; d Department of Radiology, Afyonkarahisar Health Sciences University, Afyon, Turkey

**Keywords:** Acne vulgaris, isotretinoin, semen parameters, varicocele

## Abstract

**Purpose:**

Vitamin A has multiple functions in the human body, being involved in growth, epithelial differentiation, vision, immune function and reproduction. While normal spermatogenesis is influenced by several factors, it requires vitamin A. Systemic isotretinoin is a vitamin A derivative that is used in the treatment of many dermatological diseases, especially acne vulgaris (AV). There is limited research on the changes in semen parameters after systemic isotretinoin therapy in humans. Our study investigates the presence of varicoceles in patients undergoing systemic isotretinoin therapy for AV and examines whether there were any changes in the semen parameters before and after treatment.

**Methods:**

Included in the study were 46 men patients who were scheduled for systemic isotretinoin therapy for AV. Before treatment, the patients underwent a physical examination and ultrasonography for varicoceles assessment. The patients underwent spermiogram before treatment and after 6 months of treatment. The spermiogram assessments included semen volume, sperm concentration, total sperm count, progressive motility, viability and sperm morphology.

**Results:**

After treatment, there was an increase in semen volume, sperm concentration, total sperm count, progressive motility and vitality from the pre-treatment values, but a deterioration in the sperm morphology (*p* < .05). Comparing patients with and without varicoceles revealed more changes in semen parameters after treatment in those with varicoceles. There was a statistically significant difference in sperm concentration (*p* < .001).

**Conclusions:**

Systemic isotretinoin therapy negatively affects sperm morphology, but has positive effect on other semen parameters, and these changes in semen parameters occur more frequently in patients with varicoceles.

**KEY MESSAGES**
- Acne vulgaris is a very common disease and systemic isotretinoin is used as the most effective agent in its treatment.
- Systemic isotretinoin positively affects semen parameters except sperm morphology.
- Changes in semen parameters are more common in patients with varicocele.

## Introduction

Vitamin A has multiple functions in the human body, being involved in growth, epithelial differentiation, vision, immune function and reproduction [1]. Retinoic acid (RA), a vitamin A metabolite, is critical for oocyte and sperm production in mammals. The germ cells in the foetal ovary initiate meiosis when exposed to RA, while those in the foetal testis are protected from RA and do not initiate meiosis. After birth, spermatogonial stem cells initiate spermatogenesis through periodic RA [2], and so males need dietary retinoids or vitamin A for spermatogenesis [3]. It is known that blood–testis barrier connections and RA signals play an important role in the spermatogenesis process [4]. There are limited studies investigating the effect of retinoids and vitamin A on spermatogenesis in humans. Most studies are conducted on rats due to their similarities to humans [5].

Acne vulgaris (AV) is a chronic skin condition of pilosebaceous follicles that is most common in adolescents, affecting approximately 85% of young people [6]. The pathogenesis of acne involves excessive sebum production, growth of bacteria such as *Cutibacterium acnes* or *Staphylococcus epidermidis*, abnormal follicular keratinization and inflammation [7,8]. AV is treated with topical (retinoids and antibiotics) and systemic (retinoids, antibiotics and hormones) therapies [9]. Systemic isotretinoin is a vitamin A derivative that is widely used for the treatment of many dermatological diseases, especially AV [10]. Isotretinoin inhibits the activity of the sebaceous glands and has anti-inflammatory and immunomodulatory properties. As an effective treatment for moderate to severe AV, systemic isotretinoin is the first choice of treatment [11]. Studies of male fertility and the use of isotretinoin, however, are limited and include a small number of patients [12–17].

According to WHO, the lower limit values for semen parameters were determined as 1.4 ml for semen volume, 16 × 10^6^/ml for semen concentration, 30% for progressive motility, 54% for viability and 4% for sperm morphology. It has been reported that there are spermiogram anomalies in measurements below these values and this situation poses a risk for infertility [18]. The primary and correctable cause of male infertility is varicoceles. It is believed that oxidative stress (OS) induced by a varicocele result in a deterioration in sperm parameters [19]. The administration of isotretinoin to sperm samples of men with varicoceles has been shown to activate antioxidant systems and reduce oxygen free radicals (OFRs) [20]. The present study investigates clinical and subclinical varicoceles in male patients receiving systemic isotretinoin for AV, examining the changes in sperm parameters before and after therapy and investigating the effect of systemic isotretinoin therapy on semen parameters.

## Materials and methods

### Ethics approval and consent to participate

This study was approved by the Afyonkarahisar Health Science University ethics committee (AFSU 2011-KAEK-2/2020/71) and was conducted in accordance with the ethical standards of the Declaration of Helsinki. Written informed consent was obtained from all study participants.

### Patients and study design

This prospective and observational study was conducted by the Departments of Urology, Dermatology, Radiology, and Histology & Embryology between 1 February 2021 and 1 June 2022. The study included 46 male patients over the age of 18 years who were scheduled for systemic isotretinoin therapy for AV and who volunteered to participate in the study. Patients under the age of 18 years, female patients, those who did not consent to participate in the study, those with a history of systemic drug therapy in the past three months, those with tobacco and alcohol use, those with systemic diseases, those with a previous testicular surgery and those diagnosed with infertility were excluded from the study. Patients with endocrine disease that may affect semen parameters, and patients who received radiotherapy and chemotherapy were not included in the study. Subsequently, 13 patients who discontinued isotretinoin therapy and one patient with azoospermia detected on the first spermiogram were excluded. The male patients scheduled for systemic isotretinoin therapy for AV underwent a testicular examination performed by an urologist before the study to assess the presence of varicoceles. A testicular Doppler ultrasonography was performed on the patients by a radiologist. Testicular size, presence of varicoceles, testicular vein diameter and reflux flow were recorded. Varicocele was defined as an increase in vein diameter of more than 2.5 mm at rest and more than 1.0 mm after valsalva. Semen samples were collected from the patients after 3–5 days of sexual abstinence and examined within 30 min. Viability parameters were evaluated by a specialist histologist using a macler slide under a light microscope. In addition, morphological anomalies were evaluated with diffquick staining. WHO criteria were followed when examining semen parameters [18,21]. Each patient was treated with 0.5–1.0 mg/kg oral isotretinoin up to a cumulative dose of 120 mg/kg. After 6 months of therapy, the patients underwent a further spermiogram, the results of which were assessed by a single histologist/embryologist. The specialist performing the sperm assessment was unaware of which patient gave the sample and when the sample was collected (before or after therapy). Changes in spermiogram parameters before and after therapy, and the effect of varicoceles on the results were evaluated.

### Statistical analysis

The statistical analysis of the study data was performed in a computer environment using IBM SPSS Statistics (version 20.0, IBM Corp., Armonk, NY). The normality of the variables was analysed with a Kolmogorov–Smirnov (K–S) test; and pairwise comparisons were made with Student’s *t*-test for normally distributed data and a Mann–Whitney *U*-test for non-normally distributed data. Pre-treatment and post-treatment parameters were compared with a paired *t*-test for normally distributed data and a Wilcoxon test for non-normally distributed data. The results were considered statistically significant at *p* < .05.

## Results

The mean age of the patients was 22.76 ± 5.02 (18–38) years, the mean body mass index (BMI) was 23.64 ± 3.76 (15.96–33.91) and the mean disease duration was 61.04 ± 55.86 (6–240) months. The mean global acne score was 30.43 ± 4.92 (21–44). Acne severity was moderate in 21 (45.7%) patients and severe in 25 (54.3%) patients. There were 38 (82.6%) single and eight (17.4%) married patients. Varicoceles were found in six (13%) patients on examination and ultrasonography, while six (13%) patients had subclinical varicoceles not detected on examination. No varicocele was detected on examination and ultrasonography in 34 (74%) patients. In the ultrasonographic examination performed on the patients before the study, the mean testicular volume was calculated as 11.75 ± 3.50 mm^3^ in the right testis and 13.66 ± 3.54 mm^3^ in the left testis in patients with varicocele. In patients without varicocele, the right testis was 12.30 ± 2.49 mm^3^ and the left testis was 11.19 ± 2.28 mm^3^. There was no statistical difference between the groups in terms of right and left testis volumes (*p* = .078, *p* = .068, respectively).

The mean sperm volume was 2.45 ± 1.30 ml before therapy and 3.16 ± 1.63 ml after therapy, revealing a statistically significant increase (*p* = .002). The mean sperm concentration was 21.02 ± 2.10 million/ml before therapy and 22.85 ± 2.32 million/ml after therapy, revealing a statistically significant increase (*p* = .001). The mean total sperm count was 51.45 ± 28.30 million before therapy and 73.87 ± 44.18 million after therapy, revealing a statistically significant increase (*p* < .001). The mean percentage of progressive sperm motility was 35.67 ± 8.71 before therapy and 38.46 ± 6.68 after therapy, revealing a statistically significant increase (*p* = .008). The mean percentage of viability was 72.22 ± 17.67 before therapy and 78.22 ± 13.92 after therapy, revealing a statistically significant increase (*p* = .003). The mean percentage of normal morphology was 9.37 ± 0.77 before therapy and 8.70 ± 0.99 after therapy, revealing a statistically significant decline (*p* < .001). The spermiogram results of all patients participating in the study before and after treatment are given in Table 1.

**Table 1.** Spermiogram parameters before and after systemic isotretinoin treatment.

Parameters	Before treatment (*n* = 46)	After treatment (*n* = 46)	*p*
Semen volume (ml)^a^	2.45 ± 1.30	3.16 ± 1.63	**.002**
Sperm concentration (×10^6^/ml)^a^	21.02 ± 2.10	22.85 ± 2.32	**.001**
Total sperm count (×10^6^/ejaculate)^a^	51.45 ± 28.30	73.87 ± 44.18	**<.001**
Progressive motility (%)^a^	35.67 ± 8.71	38.46 ± 6.68	**.008**
Vitality (live spermatozoa, %)^a^	72.22 ± 17.67	78.22 ± 13.92	**.003**
Sperm morphology (normal forms, %)^a^	9.37 ± 0.77	8.70 ± 0.99	**<.001**

^a^Mean ± standard deviation. Statistically significant data are written in bold in tables 1 and 2. *p*<0.05 was considered significant.

When the spermiogram parameters of 12 (26%) patients with clinical and/or subclinical varicoceles and 34 (76%) patients without varicoceles were compared, the increase in sperm concentration after therapy was found to be statistically significantly higher in the varicocele group (*p* < .001). While the increase in ejaculate volume, total sperm count, sperm progressive motility and vitality was higher in the varicocele group than in the non-varicocele group, the difference was statistically insignificant (*p* = .34, *p* = .51, *p* = .188 and *p* = .179, respectively). After therapy, a deterioration in sperm morphology was noted both in the varicocele and non-varicocele groups, but there were no statistical differences in the groups (*p* = .744). Table 2 presents an assessment of differences in semen parameters before and after therapy in the varicocele and non-varicocele groups.

**Table 2.** Spermiogram values before and after treatment in the groups with and without varicocele.

Parameters		Varicocele (12)	Non-varicocele (34)	*p*
Semen volume (ml)^a^	Before After	2.60 ± 1.29 3.71 ± 2.31	2.39 ± 1.32 2.97 ± 1.30	.34
Sperm concentration (×10^6^/ml)^a^	Before After	20.00 ± 1.95 25.25 ± 0.87	21.38 ± 2.06 22.00 ± 2.06	**<.001**
Total sperm count (×10^6^/ejaculate)^a^	Before After	51.56 ± 24.67 94.13 ± 60.34	51.42 ± 29.82 66.72 ± 35.31	.051
Progressive motility (%)^a^	Before After	36.17 ± 9.74 40.67 ± 8.52	35.50 ± 8.47 37.68 ± 5.85	.188
Vitality (live spermatozoa, %)^a^	Before After	73.83 ± 16.15 82.00 ± 17.05	71.65 ± 18.37 76.88 ± 12.66	.179
Sperm morphology (normal forms, %)^a^	Before After	9.42 ± 0.79 8.75 ± 0.97	9.35 ± 0.77 8.68 ± 1.01	.744

^a^ Mean before and after therapy ± standard deviation. Statistically significant data are written in bold in tables 1 and 2. *p*<0.05 was considered significant.

## Discussion

The present study reveals all post-treatment semen parameters other than morphology to be positively affected when compared to pre-treatment values in patients receiving systemic isotretinoin therapy, and the changes are more pronounced in patients with varicoceles.

Infertility affects 15% of the global population [22], and occurs in both males and females. While 50% of infertility cases are due to both male and female factors, a solely male factor accounts for 25% of cases [23]. Varicoceles are regarded as the primary and correctable cause of male infertility, although 45–60% of men with varicoceles have normal spermiogram values. It is believed that OS induced by varicoceles leads to a deterioration in sperm parameters [19]. Similarly, OS and OFRs are believed to be harmful to sperm, and account for 30–80% of subfertility cases [24]. Hurtado de Catalfo et al. have reported OS products in the semen sample and blood of men with varicoceles to be higher than in those without varicoceles, and these values returned to normal after varicocelectomy [25]. A reduction was identified in OFRs in semen samples collected from men with varicoceles after the administration of isotretinoin [20].

The negative effects of OS due to varicoceles on sperm parameters and the reduction of OFRs by isotretinoin added to the ejaculates of men with varicoceles suggest that systemic isotretinoin may be effective in reducing OS in the testes. Similarly, Vogt and Ewers reported increased sperm counts and motility, and deteriorated morphology after systemic isotretinoin therapy in patients with cryptorchidism, hypogonadism and varicocele, while there was no change in sperm parameters in patients without testicular pathology [13]. Çınar et al. reported increased sperm counts, vitality and motility and declined sperm morphology in patients receiving isotretinoin when compared to the pre-treatment values [16]. The studies by Török et al. and Parsch et al. including a limited number of patients reported isotretinoin therapy to have no effect on spermiogram parameters [12,26], although these studies did not mention the presence of any testicular pathology that might affect spermiogram values in the sample. Amory et al. reported that fertility increased after isotretinoin treatment in oligoasthenozoospermic and azoospermic patients in two recent studies [17,27].

A significant increase in ejaculate volume, sperm concentration, total sperm count, vitality and progressive motility was noted after therapy when compared to the pre-treatment values in patients receiving systemic isotretinoin therapy in the present study. When we compared pre-treatment and post-treatment spermiogram values between patients with and without varicoceles, those with varicoceles were found to have a statistically significantly higher increase in sperm concentration and a greater significantly insignificant increase was recorded in ejaculate volume, total sperm count, progressive sperm motility and vitality. The findings of the present study suggest that isotretinoin therapy in therapeutic dose has a positive effect on semen parameters, and that this is greater in the varicocele group.

Recently, Yokota et al. reported a deterioration in sperm morphology in mice after exposure to high-dose vitamin A [28]. Vogt et al. and Çınar et al., on the other hand, reported that isotretinoin therapy in therapeutic doses in humans led to a decline in sperm morphology [13,16]. The present study, similar to the results of previous studies, revealed a decrease in the normal sperm morphology after therapy, although not at a level that would negatively affect fertility. The negative effect of systemic isotretinoin therapy on sperm morphology seems to be a topic that warrants further investigation.

There are some limitations to this study. First, it was conducted in a single centre and with a limited number of patients due to the significant decrease in the number of patients admitted for AV due to the COVID-19 pandemic. The patients were grouped based on the presence of varicoceles before therapy, and the comparison was made with a limited number of patients with varicoceles due to the low number of patients. These limitations could be eliminated through the involvement of a higher number of patients, so multicentre studies with more patients are recommended to support the findings of the present study.

Our study found systemic isotretinoin therapy to have a positive effect on semen parameters, and the effect was even more pronounced in patients with varicoceles. The reason for the decline in sperm morphology after systemic isotretinoin therapy warrants further investigation.

## Data Availability

I accept the sharing conditions data policy upon reasonable request.
